# The Anal Reflex

**Published:** 1892-01

**Authors:** 


### THE ANAL REFLEX.

Rossolimo says this reflex consists in a contraction of the anal sphincters in a response to a stimulation to the skin and mucous membrane of the anus. It is invariably present in man in health. The branches of the inferior hemorrhoidal, pudendal and perineal nerves, on which this reflex depends, are connected with the third and fourth roots of the sacral plexus, which spring from nerve cells in the conus medullaris. This reflex can be obtained in the dog as well as in man, and Rossolimo cut the spinal cord across at different levels from above downwards; whenever the lumbar enlargement was cut across at the level of entry of the third sacral nerve, the anal reflex suddenly disappeared, from which it follows that the cells of the spinal cord which are connected with this reflex are situated in the third quarter of the lumbar enlargement, reckoning from above downwards. In another series of experiments the lumbar enlargement was exposed and the sacral roots were cut one at a time. By this means it was proved that the anal reflex depended upon the integrity of the third and fourth sacral roots. This reflex, therefore, has its seat in the cord lower than any other reflexes. To obtain the anal reflex the patient may be either standing, the operator separating the glutei, or lying on his side, with the legs drawn up. The skin and mucous membrane of the anus may be stimulated by stroking with a pin, a feather, a piece of paper or some suitable object. The reflex is shown by a contraction of the sphincter and ani externus and if it is very strong there is a drawing in of the whole anus and even sometimes a contraction of the glutei. In women the testing of this reflex may be conveniently combined with a gynæcological examination. The author has examined this reflex in a great many conditions and he comes to the following conclusions: It is increased in some cases of neurasthenia, in cases of myelitis high up in the cord, and in conditions in which there is a general exaltation of sensations. It is lost in multiple neuritis affecting the sacral plexus, in some cases of tabes and in myelitis of the lower part of the cord and in these cases there is generally also anæsthesia of the rectum, anus and urethra. It remains normal in functional derangements of the bladder, the rectum and the sexual apparrtus.—*Boston Med. and Surg. Jour.—Alienist and Neurologist.*

